# Shaving

**Published:** 1871-06

**Authors:** 


### SHAVING.

One of the most disgusting face diseases, as well as tedious of cure, is often communicated to gentlemen by being shaved in a barber's shop with a razor which has just been used on the face of a man who had the malady. It ought to be a very urgent necessity which should induce us to go into a strange shop for a shave; better go unshaven for a year, than to run the risk of a disease which it sometimes takes a year to cure. The cause of the malady is a vegetable product which sprouts up by millions around the root of each particular hair, causing a filthy yellow matter, which scabs and scales, and seems to renew itself, indefinitely long. This vegetable growth multiplies itself, and scatters its seeds by millions in a very short time. But there is an infallible preventive, costing nothing, yet the reader won't use it, not one in a hundred, simply from a reckless perversity, peculiar to the animal, man. If the razor is dipped in hot water, carefully wiped, and dipped in hot water again, the contagion is impossible, because the hot water scalds the life out the seed, as it would out of a grain of wheat, or any other farm product.

